# Long-Term Over-Expression of Neuropeptide Y in Hypothalamic Paraventricular Nucleus Contributes to Adipose Tissue Insulin Resistance Partly via the Y5 Receptor

**DOI:** 10.1371/journal.pone.0126714

**Published:** 2015-05-18

**Authors:** Min Long, Jiyin Zhou, Dandan Li, Lu Zheng, Zihui Xu, Shiwen Zhou

**Affiliations:** 1 Department of Endocrinology, Xinqiao Hospital, Third Military Medical University, Chongqing, 400037, P.R. China; 2 Base for Drug Clinical Trial, Xinqiao Hospital, Third Military Medical University, Chongqing, 400037, P.R. China; 3 Department of hepatobiliary surgery, Xinqiao Hospital, Third Military Medical University, Chongqing, 400037, P.R. China; Monash University, AUSTRALIA

## Abstract

Intracerebroventricular injection and overexpression of Neuropeptide Y (NPY) in the paraventricular nucleus (PVN) has been shown to induce obesity and glucose metabolism disorder in rodents; however, the underlying mechanisms are still unclear. The aim of this study was to investigate the mechanism contributing to glucose metabolic disturbance induced by NPY. Recombinant lentiviral NPY vectors were injected into the PVN of rats fed a high fat (HFD) or low-fat diet. 8 weeks later, *in vivo* intravenous glucose tolerance tests and euglycemic-hyperinsulinemic clamp revealed that insulin resistance of adipose tissue were induced by NPY overexpression with or without HFD. NPY increased food intake, but did not change blood glucose, glycated hemoglobin A1c (HbA1c) or lipid levels. However, NPY decreased the expression of pGSK3β, PI3K p85 and pAKT^Ser473 ^in adipose tissue of rats. *In vitro*, 3T3-L1 adipocytes were treated with NPY, NPY Y1 and Y5 receptor antagonists. Glucose consumption and 2-deoxy-D-[^3^H] glucose uptake were partly inhibited by NPY, while a decrease in PI3K-AKT pathway signaling and a decreased expression of pGSK3α and pGSK3β were observed. Nevertheless, a Y5 receptor antagonist (L-152,804) reversed the effects of NPY on glucose uptake and consumption. These data suggest that long-term over-expression of NPY in PVN contributes to the establishment of adipose tissue insulin resistance, at least partly via the Y5 Receptor.

## Introduction

Neuropeptide Y (NPY) is one of the most common peptides in the brain and is an abundant neurotransmitter in the peripheral sympathetic nervous system (SNS). NPY has been shown to play a role in energy metabolism, appetite regulation, cardiac rhythm, blood pressure, smooth muscle contraction and relaxation [1]. Cumulative evidence also suggests that NPY acts as a metabolic signal that may contribute to obesity, hyperinsulinemia, and hyperglycemia [2, 3]. Intracerebroventricular injection of NPY and overexpression of NPY in the paraventricular nucleus (PVN) of the hypothalamus, for example, have been shown to temporarily increase food intake and promote insulin release [4, 5].

The activity of NPY in cellular metabolism appears to be mediated through its ability to bind the transmembrane domain G protein coupled receptors NPY Y1–Y5 [6]. These receptors are found in a broad array of tissues including those involved in metabolism, like adipose tissue and liver. There is evidence suggesting that NPY influences metabolic function in peripheral tissue mostly via Y1 and Y5 receptor signaling [7]. However, in a stress-induced obesity model, NPY induction in fat correlated with insulin resistance that could be attenuated by blockage of the Y2 receptor [8]. The mechanism by which NPY contributes to insulin resistance in adipose tissue, therefore, is not well understood.

In this study we investigated the molecular mechanisms of NPY that contribute to peripheral insulin resistance. Our hypothesis was that NPY contributes to peripheral insulin resistance in adipose tissue via the Y5 receptor. To test this hypothesis, we created an insulin resistant *in vivo* model by injecting NPY in the PVN of the hypothalamus in rats. Eight weeks after injection, euglycemic-hyperinsulinemic clamp and intravenous glucose tolerance tests confirmed that the rats had developed insulin resistance in adipose tissue. As the main glucose metabolism pathway, PI3K-AKT signaling changes in adipose tissue were assessed in our study. Glycogen synthase kinase 3 (GSK3) α and β were also detected, since there is evidence suggesting that GSK3 contributes to the induction by insulin resistance independently of insulin receptor signaling or PI3K-AKT activity [9]. NPY Y1 and Y5 receptor antagonists were used to unravel the mechanism disorders induced by NPY in adipocytes.

## Methods and Procedures

### Reagents and antibodies

Dexamethasone, 3-isobutyl-1-methylxanthine (IBMX), bovine insulin, human NPY, and 2-deoxy-D-glucose were purchased from Sigma-Aldrich (St Louis, MO, USA). The Y5 receptor antagonist L-152,804 was purchased from Tocris Bioscience (Bristol, UK). The Y1 receptor antagonist BIBP-3226 (Diphenylacetyl-D-Arg-4-hydroxybenzylamide) was purchased from Bachem (San Carlos, CA, USA). 2-deoxy-D-[^3^H] glucose (2-[^3^H] DG) was obtained from Amersham Life Sciences (Buckinghamshire, UK).

Antibodies for immunoblot and immunofluorescence assays included: anti-NPY, anti-GSK3α, anti-GSK3β, anti-pGSK3α^Ser21^, anti-pGSK3β^Ser9^ (Santa Cruz Biotechnology, CA, USA); anti-PI3K, anti-PI3K p85, AKT, anti-pAKT^Ser473^ (Cell Signaling Technology, MA, USA). Secondary antibodies conjugated to HRP and Alexa Fluor dyes for immunoblotting and immunofluorescence were purchased from Life Technologies (Grand Island, NY, USA). Antibodies were used according to manufacturer's instructions.

### Animals

6-week old male Sprague-Dawley rats (240–260 g) were purchased from the Research Institute of Surgery Experimental Animal Center of the Third Military University (Chongqing, China) and housed individually. Initially, all rats were maintained under a controlled environment (temperature 20 ± 3°C, humidity 60 ± 5%, 12 h dark-light cycle) with regular chow consisting of 5% fat, 55% carbohydrate, 23% protein, 7% ash and 10% fiber with a total caloric value of 3.2 kcal per gram. Food and water were available *ad libitum*. Rats were used in the experiment at 9 weeks of age (290–310 g). At this time, rats were fed either the regular chow (low-fat diet, LFD) or a high-fat diet (HFD), consisting of 50% fat, 17% carbohydrate, 25% protein, 3% ash and 5% fiber with a total caloric value of 4.7 kcal per gram. Rats were handled and cared for according to the Guide for the Care and Use of Laboratory Animals, and all procedures were approved by the Ethics Scientific Committee of the Third Military Medical University.

### Lentivirus NPY production and hypothalamic PVN injection

The chemically synthesized rat NPY gene (RefSeq ID: NM_012614) was cloned into the lentiviral vector pUbi-IRES-Cherry with Agel and Nhel restriction enzymes cutting sites, the final commercial recombinant lentiviral particles containing NPY (LV-NPY-Cherry) were purchased from Shanghai GeneChem Co., Ltd. China. The empty lentiviral expression vector pUbi-Cherry (LV-Cherry) was used as a negative control. All lentivirus batches used for experiments had comparable titers ranging from 2×10^8^ to 3×10^9^ transducing units (TU)/mL. Viral suspensions were stored at -80°C until use and were briefly centrifuged and kept on ice immediately before they were injected into the PVN of rats.

At the age of 9 weeks, rats were anaesthetized with an intraperitoneal injection of sodium pentobarbital (Sigma, 36 mg/kg) and placed on a stereotaxic frame (David Kopf Instruments, Tujunga, CA) connected to a nanoliter syringe pump. After exposure of the skull surface, a burr hole was made in the skull, and the nuclear injection of LV-NPY-Cherry or LV-Cherry was carried out using a 10 μl syringe (Hamilton, Switzerland). The rats were allocated randomly to each of the indicated treatment groups and received LFD + LV-Cherry injection (LFD), HFD + LV-Cherry injection (HFD), LFD + LV-NPY Cherry injection (LFD+NPY), or HFD + LV-NPY Cherry injection (HFD+NPY). Each rat (n = 8 per group) was subjected to two injection sites, chosen according to the Rat Brain Atlas [10] as follows: point 1, 1.8 mm posterior to the bregma, 0.5 mm left lateral, 8 mm deep; point 2, 1.8 mm posterior to the bregma, 0.5 mm right lateral, 8 mm deep. 2 μL of lentiviral suspension containing 2 × 10^8^ TU/mL was injected in each point at a rate of 0.2 μL/min. After completion of the injection, the needle was left in place for 5 min before withdrawal to ensure lentiviral diffusion into the tissue. Rats received an injection of 0.05 mg/kg buprenorphine (Schering-Plough, Maarssen, Netherlands) subcutaneously for analgesia before the surgery, and then every 8 h post-surgery for the next two days. Furthermore, rats were monitored every 12 h and allowed to recover for 7 days after surgery. 3 rats died during or after the surgery because of serious bleeding and respiratory depression, and other 3 rats received lentiviral injection to ensure the maintenance of 8 rats in every group.

### Evaluation of insulin resistance

Intravenous glucose tolerance test (IVGTT): 8 weeks after LV injection, IVGTT was conducted on unanesthetized animals. Animals were fasted overnight and a 27-gauge butterfly catheter was placed in the saphenous vein for infusion of a bolus dose of 500 mg/kg body weight of a 50% dextrose solution. Blood samples were collected from the tail at 0, 5, 10, 20, 30, 45, 60, 90, and 120 min after glucose administration.

The homeostasis model assessment (HOMA-IR) also was used to detect insulin resistance as described previously [11]. HOMA-IR (mM × μU/mL) = fasting glucose (mM) × fasting insulin (μU/mL) / 22.5.

Euglycemic-hyperinsulinemic clamps: Briefly, 8 weeks after LV injection, 3 rats per group were anesthetized with sodium pentobarbital for jugular and artery catheterization as previously described [12]. Under aseptic conditions, the right jugular vein and the left carotid artery were cannulated with PE-50 cannulas attached to a 1 mL syringe containing 5 U heparinized saline. 50 U heparinized saline were used to flush the cannulas every 24 h to avoid clotting during the recovery period. For analgesia, buprenorphine was used as described above and rats were allowed to recover for 48 h. After that, rats were fasted 8 h and the euglycemic-hyperinsulinemic clamps were performed while they were awake and unrestrained [13]. Continuous infusion of 5 mU/kg min insulin (HumulinR insulin, Eli Lilly, Indianapolis, IN) was performed to acutely increase and maintain high levels of insulin for 2 h. The arterial blood glucose concentration was measured every 10 min using a Freestyle Lite glucose meter (Abbott Diabetes Care, Alameda, CA USA) and clamped at 5–6 mM by using a variable rate of 20% glucose infusion delivered via the jugular cannula. The glucose infusion rate during the second hour of clamp (GIR_60-120_) was used as the response parameter of potency of whole body insulin action. Glucose metabolic rate in individual tissues was estimated using a technique which has been described elsewhere [14]. 2-[^3^H] DG (50 μCi) was administered as a bolus 45 min before the end of the clamp. Blood samples (100–500 μL) were harvested for plasma glucose, insulin and 2-[^3^H] DG estimations during the clamp experiment. At the end of clamps, rats were anesthetized with sodium pentobarbital (100 mg/kg), and tissues were rapidly removed and frozen in liquid nitrogen for subsequent analysis. Plasma 2-[^3^H] DG concentration and the tissue accumulation of phosphorylated 2-[^3^H] DG were estimated as described previously [15]. The glucose utilization index (Rg’), an estimate of tissue glucose uptake, was calculated as described by James et al [16].

### Basal metabolism indexes

Rats were fasted for 12 h then individually housed in metabolic cages, where food and water were available *ad libitum* for exactly 24 h. After this, rats were monitored every day for eight weeks after LV injection. Food intake, body weight, and rectal temperature were recorded once a week. At the end of experiment, the body weight and naso-anal length (cm) of the animals was measured, and the Lee index was used to assess obesity by calculating the ratio between the cube root of the body weight (g) and the naso-anal length (cm) of the animals multiplied by 10 [17, 18].

### Tissue and sample preparation

Rats were sacrificed 8 weeks after LV injection by administering sodium pentobarbital, were intracardially perfused with 4% paraformaldehyde (PFA), and the brains were removed. Blood, skeletal muscle and white adipose tissues (WAT), including subcutaneous, epididymal and retroperitoneal adipose tissue were collected prior to perfusion. Fasting venous blood was collected and serum was separated by centrifugation (2000 *x g*, 15 min), adipose tissue was isolated, weighed, and immediately frozen in liquid nitrogen. Perfused rats’ brains were dehydrated gradually with cane sugar, embedded in optimal cutting temperature (OCT) compound (Sakura Finetek, Tokyo, Japan), and stored at -80°C until they were cut into sections.

### Measurements of blood indexes

Blood glucose levels were measured using a Freestyle Lite glucose meter. Serum glycated hemoglobin A1c (HbA1c) concentrations were determined using a commercially available ELISA kit (R & D Systems, Minneapolis, MN). Insulin, triglycerides and total cholesterol concentrations were measured by radioimmunoassay and enzymatic assays [19], respectively.

### Cell culture

The mouse fibroblast cell line 3T3-L1 (Cell Bank of the Chinese Academy of Sciences, Shanghai, China) was maintained and differentiated as previously described [20]. Briefly, differentiation was induced with high glucose Dulbecco’s modified Eagle’s medium containing (DMEM) 10% fetal bovine serum (FBS), 10 μg/mL insulin, 1 μM dexamethasone, and 0.5 mM IBMX. After 2 days, insulin, dexamethasone and IBMX were removed, cells were maintained in 10% FBS media with 10 μg/mL insulin for another 2 days, then the insulin was removed, and cells were cultured in high glucose DMEM with 10% FBS until the day of experiments (days 7–8). NPY, insulin, Y5 receptor antagonist L-152,804 and Y1 receptor antagonist BIBP-3226 were dissolved in DMSO, and then diluted in the appropriate test solvent.

### Glucose consumption and glucose uptake

Glucose consumption was conducted as described previously [21, 22]. The differentiated 3T3-L1 adipocytes were plated into 96-well plates, incubated with DMEM containing 0.2% bovine serum albumin (BSA) for 12 h and treated with insulin for 2 h or various concentrations of NPY for 12 h for detecting basal glucose consumption[23–25]. To test the effect of receptor antagonists on insulin-stimulated glucose consumption intervened with NPY, the adipocytes were pre-treated with L-152,804 or BIBP3226 8 h, then subsequently treated with NPY for 12 h with insulin for 2 h[26, 27]. The glucose concentration in the culture medium was determined using the glucose oxidase method [28]. The amount of glucose consumption was calculated by subtracting the glucose from the control well.

2-[^3^H] DG uptake was measured according to a method described previously [22, 29]. In brief, insulin-simulated glucose uptake was studied in differentiated monolayers. The cells were cultured in 12-well plates and starved 12 h before treatment. Then cells were pre-treated with L-152,804 or BIBP-3226 8 h and subsequently treated with NPY for 12 h and insulin for 20 min in KRH buffer (20 nM HEPES, 136 mM NaCl, 4.7 mM KCL, 1.25 mM MgCl_2_, 1.25 mM CaCl_2_, pH 7.4) at 37°C for 30 min. The cells were subsequently incubated with 2-deoxy-D-glucose (0.1 mM) and 2-[^3^H] DG (0.5 μCi/mL) for 10 min, reaction was stopped quickly with cold PBS, and cells were solubilized in 0.4 mL of 0.1 M sodium hydroxide. Radioactivity of 2-[^3^H] DG was determined in the whole cell lysates using a Beckman LS6500 scintillation counter.

### Immunofluorescence

OCT-embedded brain tissues were cut into 4 μm serial sections. Sections were incubated with 0.3% Triton X-100 at 37°C for 20 min, 5% normal goat serum at RT for 10 min, then incubated with anti-NPY primary antibody (1:100 dilution) at 4°C overnight; as a negative control, sections were incubated without primary antibody. After they were washed three times, sections were incubated with the appropriate secondary antibody directly conjugated with FITC for 1 h, stained with DAPI for 5 min at RT, then washed extensively, rinsed in ddH_2_O and mounted using gelvatol. Confocal laser imaging was performed using a Leica TCS-SP5 confocal scanning laser microscope (Leica Microsystems, Germany), and the level of NPY overexpression in PVN was quantified by ImageJ software.

### Western blotting

3T3-L1 adipocytes were plated into 6-well plates, incubated with DMEM containing 0.2% BSA for 12 h and pre-treated with 100 μM L-152,804 or 100 μM BIBP-3226 8 h, then subsequently treated with 100 nM NPY for 12 h with 100 nM insulin for 2 h. Whole cell lysates were obtained using a commercial cell protein extraction buffer (Thermo Scientific Pierce, USA) with 0.5 mM phenymethanesulfonyl fluoride (PMSF) and 10 mM Ser/Thr phosphatase inhibitor. Retroperitoneal adipose tissue was also homogenized in tissue protein extraction buffer with PMSF and phosphatase inhibitor. Samples were centrifuged at 10,000 *x g* at 4°C for 15 min, which resulted in the separation of a fat layer. The supernatants were removed making sure there was no any residual fat and centrifuged at 10,000 *x g* at 4°C for 15 min again. The resulting supernatant solutions were used for Western blot analysis. Protein concentrations were measured using a NanoDrop2000 (Thermo Scientific Pierce, USA). Equal amounts of protein (50 μg) were subjected to SDS-PAGE and transferred onto polyvinylidene fluoride (Millipore, Bedford MA) membranes. Membranes were blocked with 3% BSA/PBS-Tween20 0.1% at RT for 1 h and incubated with primary antibodies (1:500–1:1000) at 4°C overnight. The membranes were then incubated with the appropriate HRP-linked secondary antibodies at RT for 1 h. Blots were developed using an enhanced chemiluminescence method (Thermo Scientific Pierce, USA).

### Statistical analyses

For analysis of body weight, daily food intake and body temperature of rats, two-way ANOVA with repeated measures was applied with factors of group and time. Tukey's post hoc test was used when ANOVA indicated significance. One-way ANOVA was applied to analyze other indexes in *in vivo* and *in vitro* experiments. Unpaired Student’s t tests were used to compare the means of two groups in both *in vivo* and *in vitro* experiments. All results were analyzed using the GraphPad Prism software (version 6.0; Graphpad, San Diego, CA; www.graphpad.com). Statistical significance was defined as *P*<0.05.

## Results

### Constitutive overexpression of NPY in the PVN of rats

**Fig 1:**
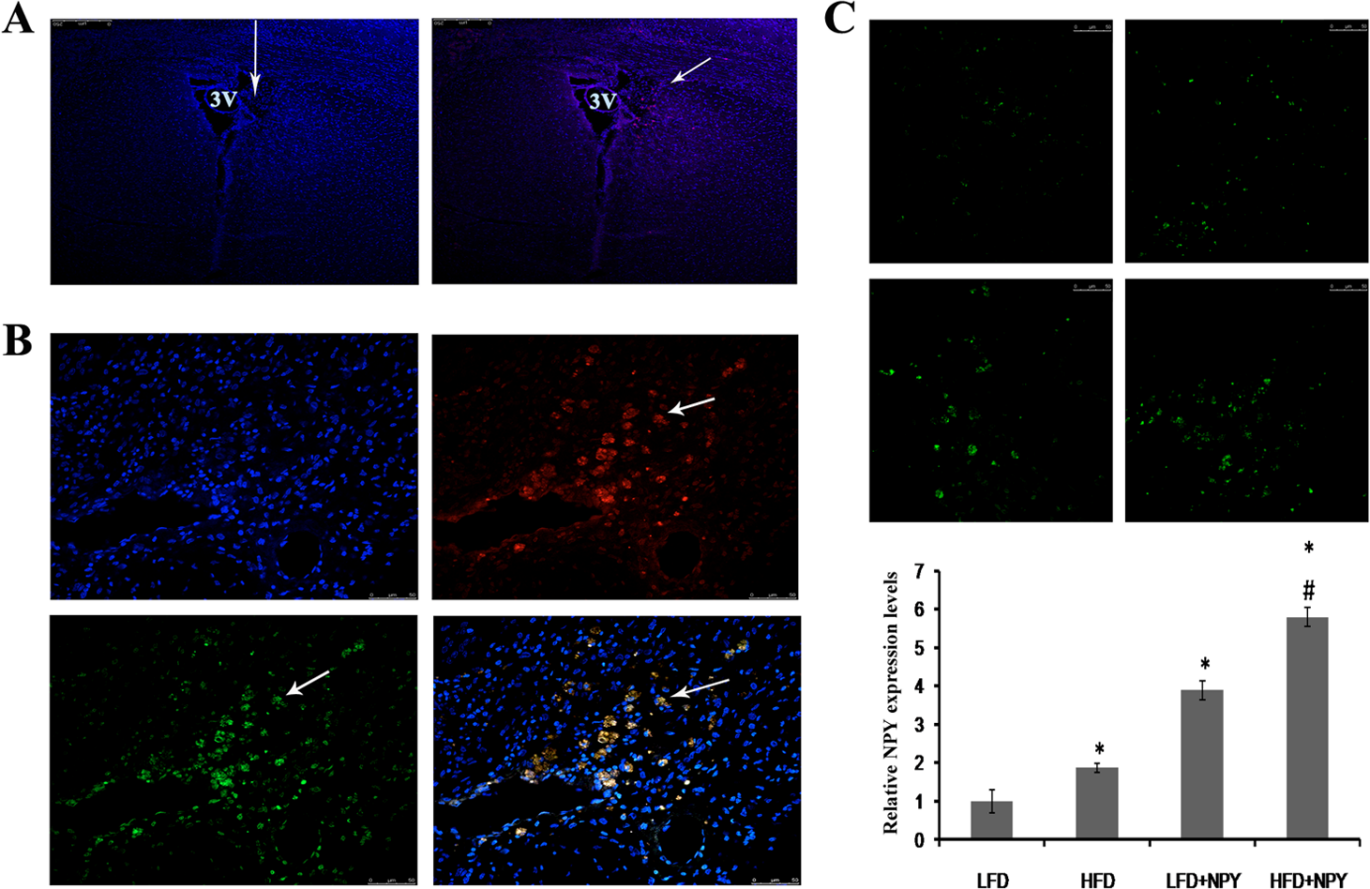
Constitutive overexpression of NPY in the paraventricular nucleus (PVN) of rats. Eight weeks after the LV-Cherry (vehicle control) or LV-NPY-Cherry injection, the expression of the vectors was measured using immunofluorescence. 2 rats were excluded because of imprecise injection. (A) The lentiviral vectors LV-Cherry or LV-NPY-Cherry were injected vertically into the PVN (left panel). The arrow shows the direction of injection and the position of the PVN in the rat brain, and the blue immunofluorescence indicate nucleus. Expression of the reporter protein Cherry (red) in PVN after injection of LV-Cherry (right panel). The arrow indicates Cherry expression in neurons. 3V: the third ventricle of cerebrum (amplification: 100x). (B) Photomicrographs of NPY and Cherry overexpression in PVN after lentivirus injection. blue immunofluorescence for nucleus, green for NPY, red for cherry, and yellow for co-localization of NPY and cherry (amplification: 400x). The arrows indicate NPY and/or Cherry expression in a single neuron of the PVN. (C) The representative images of NPY overexpression from LFD (top left), HFD (top right), LFD+NPY (left bottom) and HFD+NPY (right bottom) groups, and quantification data is shown in the bottom (n = 8) (amplification: 400x). Data are presented as means ± SEM, *P<0.5 vs. LFD; ^#^ *P*<0.5 vs. HFD.

The LV-Cherry (vehicle control) or LV-NPY-Cherry was injected into the PVN of rats. Eight weeks after injection, the expression of the vectors was measured using immunofluorescence. Rats were only included in the study when the reporter protein (Cherry) expression was located exactly in the PVN. As shown in Fig 1, both vectors were still expressed in the PVN 8 weeks after injection. Additionally, the expression of NPY and Cherry co-localized, as shown in the merged image. Comparing with LFD group, LV-NPY-Cherry injection in PVN induced a 3.89-fold increase of NPY protein expression in LFD+NPY group (*P*<0.05); and a 3.09-fold increase of NPY protein expression in HFD+NPY group (*P*<0.05) compared to HFD group. These findings suggest that we were successfully able to overexpress NPY in the PVN of rats, and overexpression of NPY can be sustained for more than 8 weeks in the hypothalamic PVN of rats by injection of recombinant lentivirus-mediated NPY particles *in situ*.

### Overexpression of NPY contributes to insulin resistance in rats

**Fig 2:**
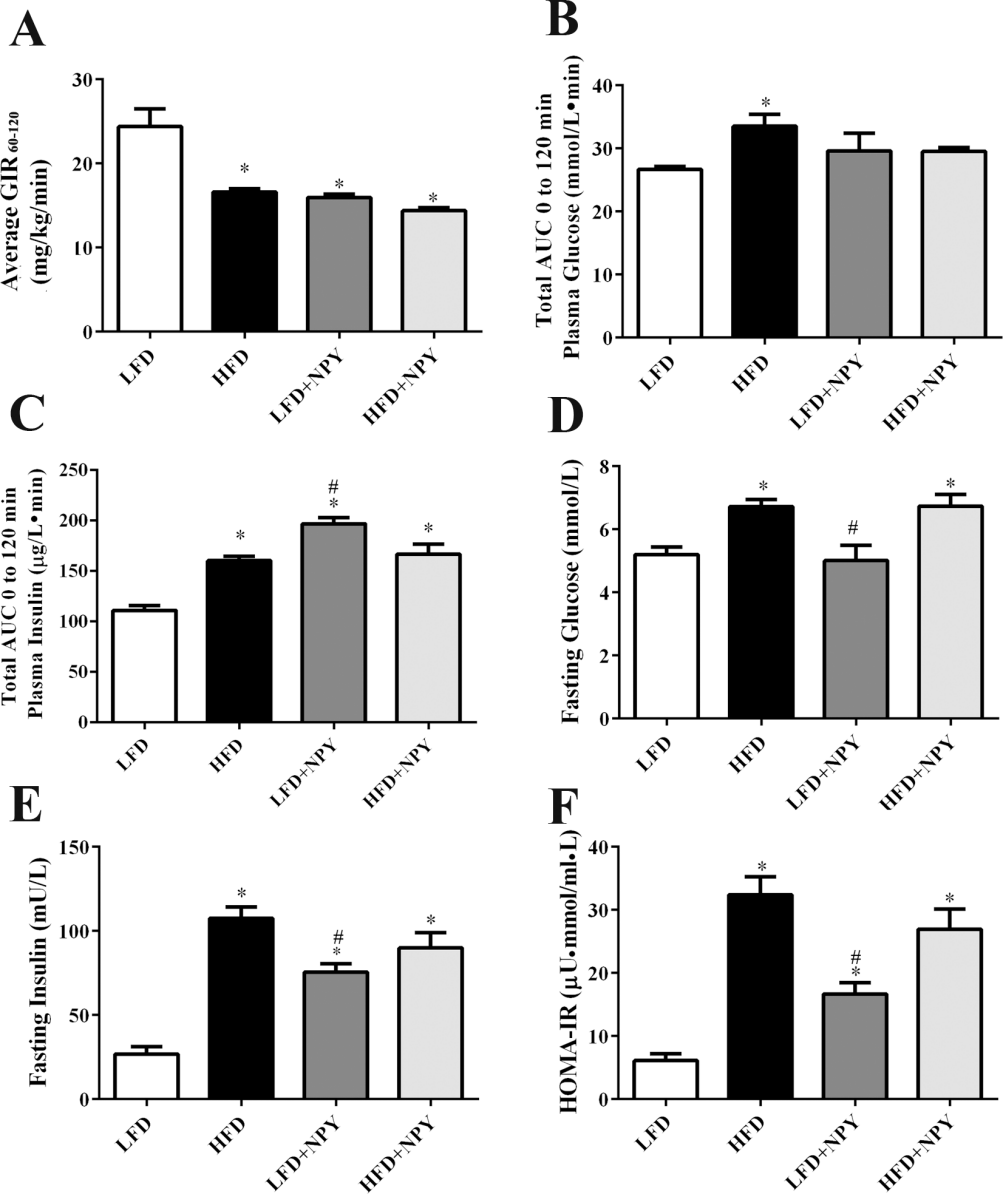
Overexpression of NPY contributes to insulin resistance in rats. Eight weeks after dietary and lentiviral intervention, insulin resistance was evaluated. (A) Average glucose infusion rate (GIR_60-120_) of the four groups (LFD, HFD, LFD+NPY, HFD+NPY) in euglycemic-hyperinsulinemic clamps (n = 3). (B) Total area under curve (AUC) of plasma glucose and (C) total area under curve (AUC) of plasma insulin during a 120 min intravenous glucose tolerance test (IVGTT) in the four groups (n = 4). (D) Fasting blood glucose, (E) fasting plasma insulin, and (F) homeostasis model-insulin resistance (HOMA-IR) (n = 8), in the four groups. HOMA-IR was calculated according to the formula: fasting insulin (μU/mL) × fasting glucose (mM) / 22.5. Data are presented as means ± SEM, *P<0.5 vs. LFD; ^#^ *P*<0.5 vs. HFD.

The euglycemic-hyperinsulinemic clamp assay showed that HFD and NPY overexpressing rats had lower GIR_60-120_ than LFD rats (Fig 2A), which indicated that peripheral insulin resistance was induced in rats fed with HFD or rats overexpressing NPY for 8 weeks. Otherwise, even though the plasma glucose levels did not increase (Fig 2B), the insulin levels measured during a 120 min intravenous glucose tolerance test (IVGTT) increased in the three groups (Fig 2C), which is consistent with the result of euglycemic-hyperinsulinemic clamp assay. Moreover, HOMA-IR test confirmed previous results, demonstrating that rats overexpressing NPY with or without a HFD develop insulin resistance (Fig 2F). Interestingly, although compared with the LFD group, the other three groups had higher fasting insulin levels (Fig 2E), a HFD did increase fasting glucose levels in both the control and NPY-overexpressing rats, but constitutive overexpression of NPY in the PVN alone did not induce obvious higher fasting glucose levels (Fig 2D).

### Overexpression of NPY induces obesity and insulin resistance in adipose tissue

**Fig 3:**
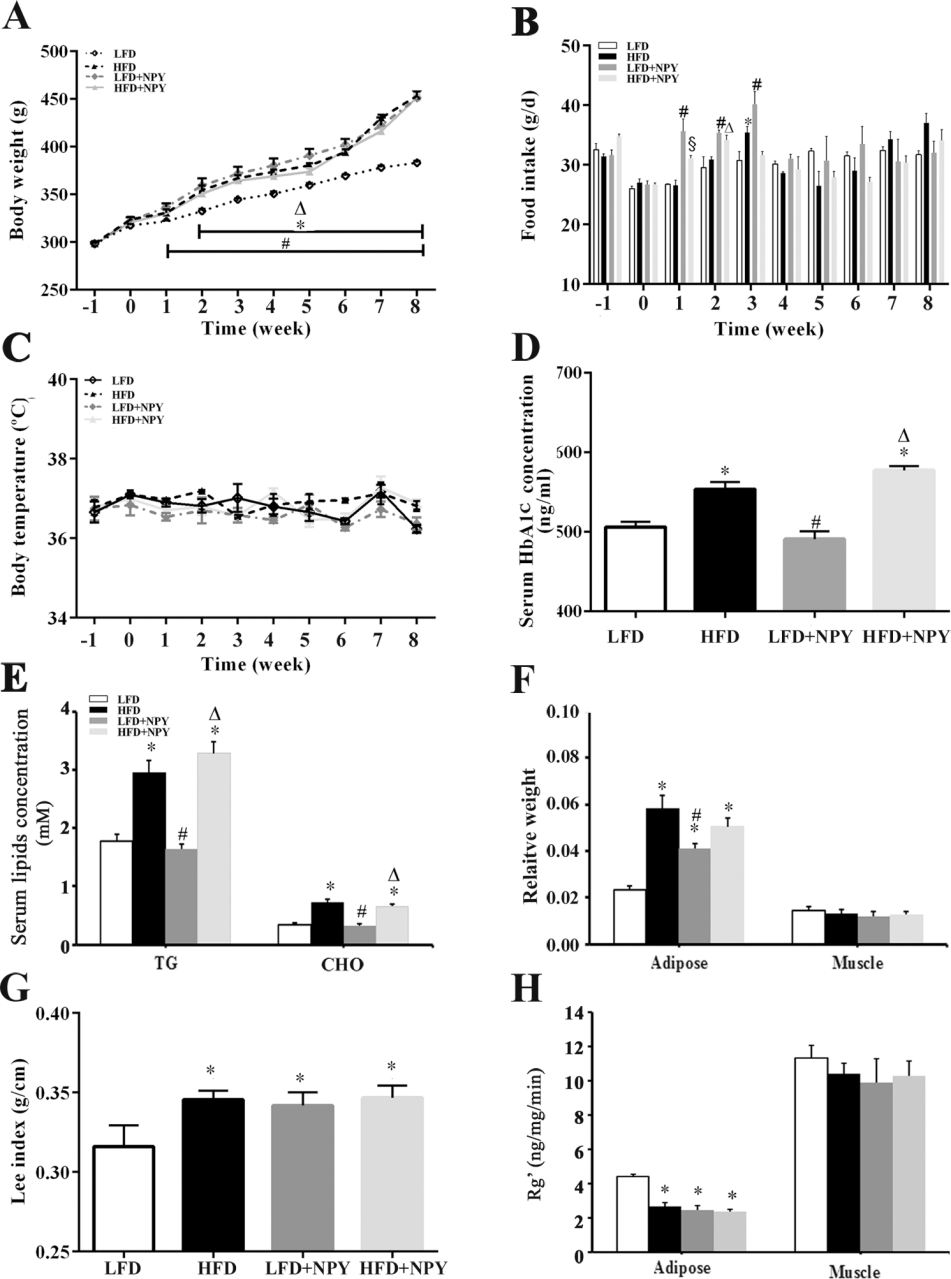
Overexpression of NPY induces obesity and decreases glucose utilization in adipose tissue. (A) Effects of HFD and NPY overexpression in PVN on body weight (n = 8), (B) food intake per day (n = 4), and (C) body temperature (n = 4). Time = 0 indicates the time of surgery. Data are presented as means ± SEM. *P<0.05, LFD vs. HFD; ^#^ *P*<0.05, LFD vs. LFD+NPY; ^∆^ *P*<0.05, LFD vs. HFD+NPY; ^§^ *P*<0.05, HFD vs. HFD+NPY across the weeks denoted by the horizontal bar. (D) Effects of HFD and NPY overexpression in PVN on serum HbA1c concentration, (E) serum triglyceride (TG) and cholesterol concentration (CHO) concentration adipose tissue, and (F) relative weight calculated as the ratio of adipose tissue and muscle weight to body weight (F), n = 8. (G) Effects of HFD and NPY overexpression in PVN on Lee index, a normal index to assess obesity, which was calculated as the ratio between the cube root of the body weight (g) and the naso-anal length (cm) of the animals multiplied by 10, n = 8. (H) glucose utilization index (Rg’) of adipose tissue and muscle, an estimate of tissue glucose utilization, n = 3. Data are presented as means ± SEM, *P<0.5 vs. LFD; ^#^ *P*<0.05 vs. HFD; ^∆^ *P*<0.5 vs. LFD + NPY group.

The body weight of HFD, LFD+NPY and HFD+NPY rats were significantly higher than those of LFD rats, especially during the last period of the experiment (Fig 3A). Noticeably, however, the food intake per day of LFD+NPY and HFD+NPY groups was greater than the LFD and HFD groups at the beginning of the experiment but leveled out by the week 4 (Fig 3B). A diet high in fat or overexpression of NPY did not alter the rats’ body temperature (Fig 3C). The serum HbA1c, triglyceride, and cholesterol concentrations were not different in the control versus NPY-overexpressing rats; however, they were significantly elevated in rats fed a HFD, and in rats overexpressing NPY who were fed a HFD (Fig 3D and 3E). This finding correlates with the results presented in Fig 2D, showing that a HFD also increases fasting glucose levels. A HFD and chronic overexpression of NPY in the hypothalamic PVN of rats increased the ratio of total adipose tissue weight to body weight (Fig 3F), which is consistent with the Lee index (Fig 3G), the indicator of obesity. These results indicated that both HFD and NPY overexpression induced obesity, but the latter didn’t increase glucose, HbA1c, or blood lipid levels. On the other hand, peripheral insulin resistance had been confirmed by the euglycemic-hyperinsulinemic clamp test and IVGTT. To further reveal the contribution of individual tissues to peripheral insulin resistance, the glucose utilization index (Rg’), an estimate of tissue glucose uptake, of adipose tissue and muscle was examined [16, 30, 31]. The results indicated that the 2-[^3^H] DG uptake of adipose tissue decreased in HFD, LFD+NPY and HFD+NPY groups, while there were not obvious differences in 2-[^3^H] DG uptake in muscle among all groups (Fig 3H).

### NPY modulates the PI3K-AKT and GSK signaling pathways

**Fig 4:**
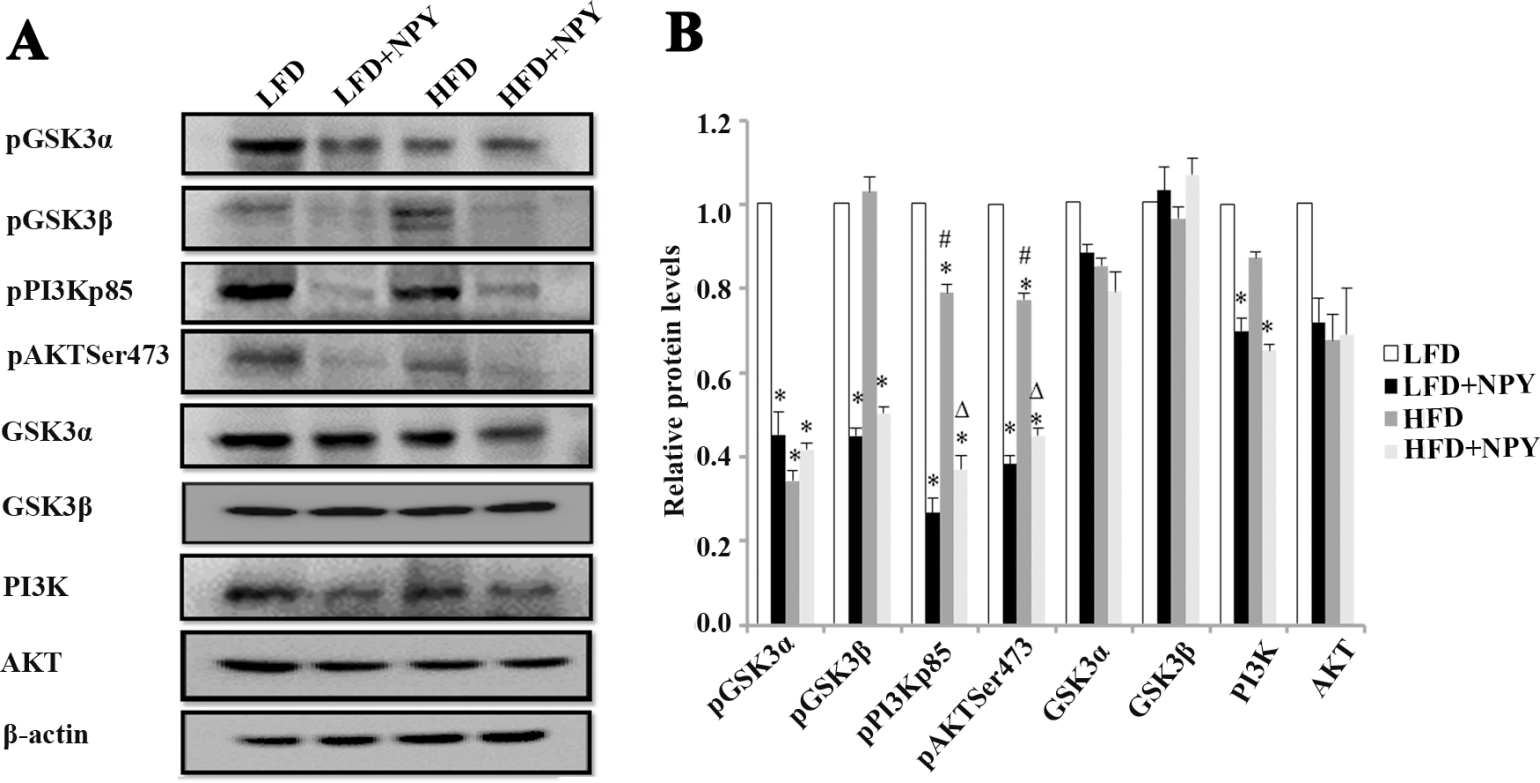
NPY modulates the PI3K-AKT and GSK signaling pathways. LV-NPY injection in the PVN for 8 weeks changed the protein expression levels of the PI3K-AKT pathway and GSK3 phosphorylation. (A) WB of pGSK3α, pGSK3β, pPI3K p85, pAKT^Ser473^, GSK3α, GSK3β, PI3K and AKT in adipose tissue of rats; (B) relative protein expression intensity normalized to β-actin. Data are presented as means ± SEM, n = 4; *P<0.5 vs. LFD; ^#^ *P*<0.05 vs. HFD; ^∆^ *P*<0.5 vs. LFD + NPY group.

Compared with rats fed a LFD, the PI3K protein levels in the adipose tissue of rats overexpressing NPY fed a LFD or HFD decreased slightly, although the AKT, GSK3β, and GSK3α protein levels did not change. Specifically, constitutive overexpression of NPY in PVN of rats dramatically decreased the phosphorylation of GSK3β, PI3K and AKT independently of the diet they were fed. However, the phosphorylation of GSK3α significantly decreased in the HFD, LFD+NPY and HFD+NPY groups (Fig 4A and 4B).

### NPY inhibits glucose consumption and 2-[^3^H] DG uptake in 3T3-L1 adipocytes via the NPY Y5 receptor

**Fig 5:**
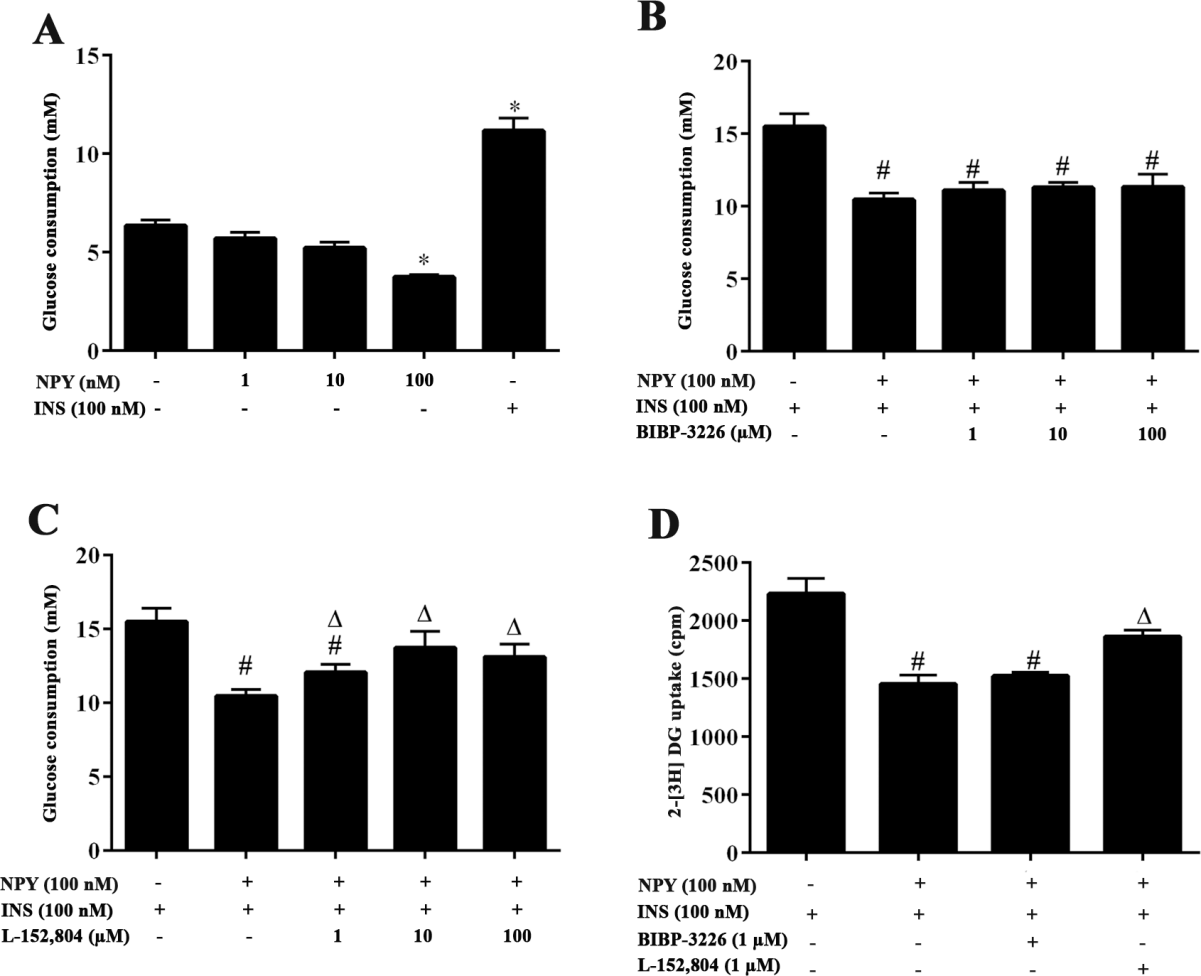
NPY inhibits glucose consumption and 2-[^3^H] DG uptake in 3T3-L1 adipocytes via the NPY Y5 receptor. (A) The effect of NPY on basal glucose consumption of 3T3-L1 adipocytes. The differentiated 3T3-L1 adipocytes were incubated with DMEM containing 0.2% BSA for 12 h and then treated with 1–100 nM NPY for 12 h or 100 nM insulin (INS) for 2 h to detect basal glucose consumption. Insulin was used as a positive control. The amount of glucose that disappeared in the medium was determined as the amount of glucose consumption. (B) The effect of Y1 receptor antagonist BIBP-3226 on insulin-stimulated glucose consumption in 3T3-L1 adipocytes treated with NPY. Cells were pre-treated with 1–100 μM BIBP-3226 for 8 h, then subsequently treated with 100 nM NPY for 12 h with 100 nM insulin for 2 h to detect insulin-stimulated glucose consumption. (C) The effect of Y5 receptor antagonist L-152,804 (1–100 μM) on insulin-stimulated glucose consumption of 3T3-L1 adipocytes treated with NPY. The cells were treated as described above. Data are presented as means ± SEM, n = 6; *P<0.5 vs. basal; ^#^ *P*<0.05 vs. INS alone; ^∆^ *P*<0.5 vs. NPY+INS. (D) The effect of Y1 receptor antagonist BIBP-3226 and Y5 receptor antagonist L-152,804 on insulin-stimulated 2-[^3^H] DG uptake in 3T3-L1 adipocytes treated with NPY. The differentiated 3T3-L1 adipocytes were pre-treated with 1 μM L-152,804 or 1 μM BIBP-3226 for 8 h and then treated with 100 nM NPY for 12 h and 100 nM insulin for 20 min in KRH buffer. The cells were subsequently incubated with 0.1 mM 2-deoxy-D-glucose (2-DG) and 0.5 μCi/ml 2-[^3^H] DG for 10 min. Radioactivity of 2-[^3^H] DG of the whole cell lysates was measured. Data are presented as means ± SEM, n = 3; ^#^ *P*<0.05 vs. INS alone; ^∆^ *P*<0.5 vs. NPY +INS.

In *in vitro* experiments we found that NPY can directly decrease the glucose uptake in 3T3-L1 adipocytes. High dose NPY (100 nM) reduced basal glucose consumption, whereas lower dose NPY (1 and 10 nM) failed to affect the basal glucose consumption in 3T3-L1 adipocytes (Fig 5A). Although NPY can also reduce the insulin-simulated glucose consumption, BIBP-3226, a NPY Y1 receptor antagonist, did not reverse the effect of NPY treatment in the adipocytes (Fig 5B). NPY Y5 receptor antagonist L-152,804 (1–100 μM) obviously reversed the restrain of NPY on insulin-simulated glucose consumption (Fig 5C). Furthermore, 2-[^3^H] DG uptake experiments demonstrated that the NPY Y1 receptor antagonist did not contribute to the insulin-simulated 2-[^3^H] DG uptake of 3T3-L1 adipocytes incubated with 100 nM NPY, whereas the NPY Y5 receptor antagonist does (Fig 5D), suggesting that the NPY Y5 receptor is responsible for the observed NPY effects.

### NPY changes the PI3K-AKT and GSK signaling pathways in 3T3-L1 adipocytes via the NPY Y5 receptor

**Fig 6:**
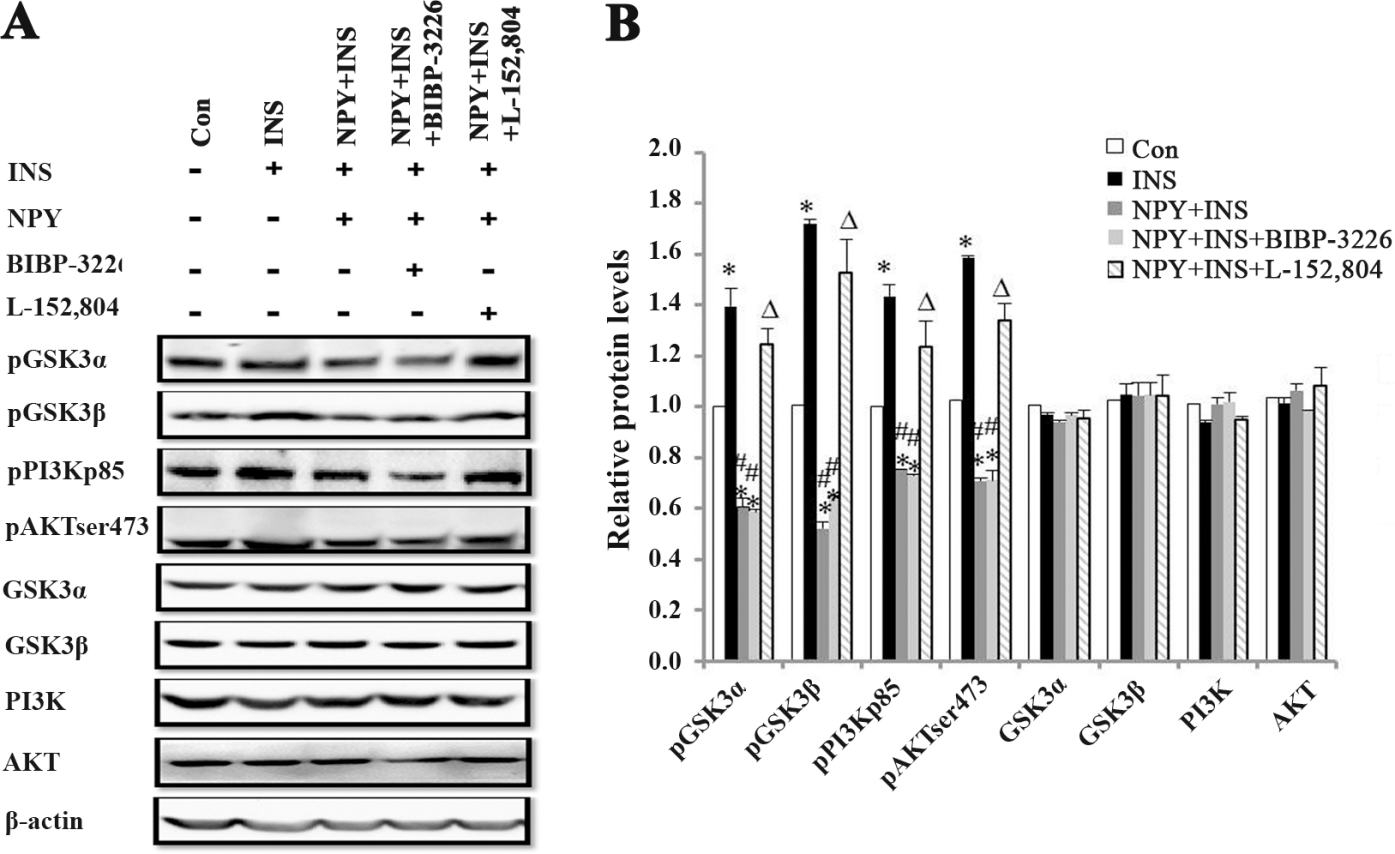
NPY changes the PI3K-AKT and GSK signaling pathways in 3T3-L1 adipocytes via the NPY Y5 receptor. The differentiated 3T3-L1 adipocytes were pretreated with 100 μM L-152,804 or 100 μM BIBP3226 for 8 h, then subsequently treated with 100 nM NPY for 12 h and 100 nM insulin for 2 h. (A) WB of pGSK3α, pGSK3β, pPI3K p85, pAKT^Ser473^, GSK3α, GSK3β, PI3K and AKT in 3T3-L1 adipocytes. (B) Relative protein expression intensity normalized to β-actin. Data are presented as means ± SEM, n = 3; *P<0.5 vs. Con; ^#^ *P*<0.05 vs. INS; ^∆^ *P*<0.5 vs. NPY + INS.

NPY inhibited the phosphorylation of GSK3α, GSK3β, PI3K and AKT significantly (Fig 6A), although NPY did not change the AKT, PI3K, GSK3α and GSK3β total protein levels in 3T3-L1 adipocytes (Fig 6B). Treatment with the NPY Y5 receptor antagonist reversed the suppression, whereas the NPY Y1 receptor antagonist did not, corroborating the previous findings that the NPY Y5 receptor is responsible for the effects of NPY in adipocytes.

## Discussion

In this study, we found that overexpression of NPY can be sustained for more than 8 weeks in the hypothalamic PVN of rats by injection of recombinant lentivirus-mediated NPY particles *in situ* with a >3-fold protein induction compared with vector injection. NPY overexpression temporarily increased daily food intake after injection and then leveled out by the week 4. A similar phenomenon was found in other comparable studies [5, 32, 33]. Tiesjema et al. have shown that NPY overexpression in PVN significantly induced daily food intake during the 6 weeks after injection of recombinant adeno-associated viral particles containing NPY cDNA in rats when allowed to eat *ad libitum* until “a certain body weight” not specified by the authors was achieved [5]. Noticeably, at the beginning of our experiments the rats’ body weights (290–310 g) were significantly higher than those of their experiment (220–250 g). This could partly explain the fact that we found an increase in food intake only up to week 3 because our heavier rats could be closer to that “certain body weight”. At the end of the experiment, the body weights of the NPY groups increased significantly compared with the LFD group, which was consistent with other experiments [5]. Moreover, the relative weight of adipose tissue and Lee index increased in HFD and NPY overexpression groups, which suggested that either HFD or long-term NPY expression in PVN result in obesity. However, we failed to find any obvious differences in body weight, adipose tissue weight, and Lee index among rats in the HFD group and those in the NPY-overexpressing groups, which might suggest that the effect of NPY on adipose tissue does not synergize with the effect caused by feeding a HFD.

We further found both NPY overexpression in PVN and HFD can decrease the average GIR_60-120_ of euglycemic-hyperinsulinemic clamps, increase total plasma insulin AUC 0 to 120min of IVGTT, HOMA-IR and fasting insulin levels, which indicated that both NPY overexpression and HFD induce peripheral insulin resistance. To reveal the contribution of individual tissues to the peripheral insulin resistance, the glucose utilization index (Rg’) of adipose and muscle was detected. The obvious decreased in glucose utilization inadipose tissue was demonstrated in insulin resistance groups, and even there was a trend of decreased in glucose utilization in muscle. These results suggested that adipose tissue might have a higher contribution to the peripheral insulin resistance seen in HFD and NPY overexpressing rats.

In this study, insulin resistance was established in NPY overexpressing rats independently of the diet, and there was no difference in body and adipose tissue weights between HFD and LFD+NPY groups. HFD induced higher fasting glucose levels, serum HbA1c concentration and dyslipidemia while NPY overexpression did not induce any effects on blood glucose or lipids. However, it could be difficult to conclude from the present data whether insulin resistance in the NPY overexpression group was a result of direct or indirect effects through obesity. Even in a pair-feeding study, restriction of food intake also could not eliminate the increase in adiposity induced by NPY [3]. Interestingly, a short-term NPY overexpression study suggested that NPY influences leptin and insulin secretion independently from food intake and obesity [32]. Additionally, activation of the parasympathetic nervous system by NPY [34] and the abundance of autonomic nerves in adipose tissue [35] may partly explain how NPY directly contributes to the establishment of insulin resistance in adipose tissue in rats. However, *in in vivo* experiments we also found that NPY can directly decrease the glucose uptake in adipocytes.

Our results indicate that the IR in adipose tissue might be driven by central NPY action, but it is hard to explain whether the development of IR is a direct consequence of the central or the peripheral Y5 signaling or both. More *in vivo* experiments need to be performed to explore the definite ways by whichthe central NPY affects the adipose tissue, such as throughthe sympathetic nerves, the adrenal medulla, platelets and various cell types within WAT [36]. However previous studies suggest a plausible mechanism that explains how central NPY exerts a function on WAT. It is well known that the hypothalamus is a major source of forebrain input into the SNS [37]. Immunostaining after pseudorabies virus (a transneuronal tract tracer) injection into fat revealed that PVN was one of the sites that modulated SNS outflow to the WAT [38]. Recently, the definition of hypothalamus-SNS-adipose tissue was used to clarify the feasibility of crosstalk between hypothalamus and adipose tissue [39]. Moreover, there is data that suggests that the pituitary-adrenal ensemble and other endocrine cues may be engaged by prolonged central administration of NPY [4]. It is verified that increased hypothalamic NPY inhibits sympathetic nerve system outflow and suppresses catecholamine release, mainly norepinephrine (NE) [39]. Furthermore, NPY colocalized with NE in peripheral SNS as an adrenergic cotransmitter which exerts pleiotropic activities either synergistic or antagonistic with NE [40]. Recent data also demonstrated that the release of NPY as a sympathetic neurotransmitter directly into the WAT leads to abdominal obesity with the depletion of NE in the adipose tissue [41].Moreover, the increased Y5 receptor expression in adipose tissue of obese animals and differentiated adipocytes[42–44] proved valuable cue to explain why NPY overexpression in PVN induced obesity and insulin resistance partly via Y5 receptor[45] in this experiment.

Furthermore, we also observed that the phosphorylation of PI3K and AKT was inhibited by NPY overexpression and HFD, and NPY aggravated the down regulation on PI3K-AKT pathway by HFD in adipose tissue. Recently, more evidence suggests that GSK3 contributes to the development of peripheral insulin resistance and type 2 diabetes [46, 47]. Moreover, high glucose induced insulin resistance by decreasing the phosphorylation of GSK3β, triggering the ubiquitination and degradation of insulin receptor substrate (IRS1), which did not require insulin receptor signaling or PI3K/AKT activity [9]. In the current study, NPY overexpression dramatically decreased the phosphorylation of GSK3β. However, HFD failed to induce any obvious change in the phosphorylation of GSK3β in adipose tissue, which implies that the differential phosphorylation of GSK3β may be one important factor to explain a different mechanism of insulin resistance in adipose tissue induced by NPY overexpression and HFD. NPY-induced insulin resistance of adipose tissue was consistent with dexamethasone-induced insulin resistance in muscle, which also decreased insulin-stimulated AKTand GSK3 phosphorylation, and completely blocked the ability of insulin to dephosphorylate and activate glycogen synthase without reducing expression of these proteins [48].

This study also demonstrated that high dose (100 nM) NPY can inhibit basal, insulin-simulated glucose consumption, and insulin-stimulated 2-[3H] DG uptake in 3T3-L1 adipocytes, suggesting that NPY can directly affect glucose uptake and consumptions in adipocytes. This result is consistent with other reports that found that NPY 100 nM inhibited fat metabolism in adipocytes [49, 50]. Y1 and Y5 receptors are most likely the receptors that contribute to the metabolic effects of NPY on adipocytes [49, 51, 52]. In this study, the NPY Y5 receptor antagonist L-152,804 specifically reversed the inhibition of NPY on glucose consumption and 2-[^3^H] DG glucose uptake in 3T3-L1 adipocytes. Moreover, previous studies report that L-152,804 did not show significant affinity for Y1, Y2, and Y4 receptors even at a dose of 10 mM [53]. Therefore we conclude that the effect of NPY on glucose consumption depends mostly on the Y5 receptor. In addition, our study further proved that NPY not only inhibits phosphorylation of PI3K and AKT proteins, but that it also decreases the phosphorylation of GSK3α and GSK3β protein significantly in 3T3-L1 adipocytes. Once again, the NPY Y5 receptor antagonist L-152,804 relieved the suppression, but the Y1 receptor antagonist BIBP-3226 did not contribute to this relief. However, NPY did not change the AKT, PI3K, GSK3α and GSK3β total protein levels in 3T3-L1 adipocytes.

In summary, this study has demonstrated the establishment of insulin resistance in rats’ adipose tissue, after long-term constitutive overexpression of NPY in the PVN of hypothalamus. NPY may affect glucose metabolism by modulating the phosphorylation of PI3K, AKT and GSK3 in adipose tissue and adipocytes, and it is possible that the NPY Y5 receptor contributes to glucose metabolism disorder induced by NPY. This study might provide more direct pharmacological evidence that using NPY receptors antagonists could prove beneficial in the treatment of diseases induced by NPY disorders.
